# Motor effort training with low exercise intensity improves muscle strength and descending command in aging

**DOI:** 10.1097/MD.0000000000003291

**Published:** 2016-06-17

**Authors:** Changhao Jiang, Vinoth K. Ranganathan, Junmei Zhang, Vlodek Siemionow, Guang H. Yue

**Affiliations:** a Department of Biomedical Engineering, Cleveland Clinic, Cleveland, OH; b Department of Physical Medicine and Rehabilitation, Cleveland Clinic, Cleveland, OH; c Human Performance and Engineering Research, Kessler Foundation, West Orange, NJ; d Department of Physical Medicine and Rehabilitation, Rutgers New Jersey Medical School, Rutgers University, Newark, NJ; e Key Lab of Sports Ability Evaluation and Comprehensive Research Lab of General Administration of Sports, Capital Institute of Physical Education, Beijing, China; f Graduate School, Beijing Sports University, Beijing, China.

**Keywords:** aging, maximal voluntary contraction (MVC), mental effort, motor activity-related cortical potential (MRCP), muscle strength, power of EEG frequency

## Abstract

This study explored the effect of high mental effort training (MET) and conventional strength training (CST) on increasing voluntary muscle strength and brain signal associated with producing maximal muscle force in healthy aging. Twenty-seven older adults (age: 75 ± 7.9 yr, 8 women) were assigned into 1 of 3 groups: MET group—trained with low-intensity (30% maximal voluntary contraction [MVC]) physical exercise combined with MET, CST group—trained with high-intensity muscle contractions, or control (CTRL) group—no training of any kind. MET and CST lasted for 12 weeks (5 sessions/week). The participants’ elbow flexion strength of the right arm, electromyography (EMG), and motor activity-related cortical potential (MRCP) directly related to the strength production were measured before and after training. The CST group had the highest strength gain (17.6%, *P* <0.001), the MET group also had significant strength gain (13.8%, *P* <0.001), which was not statistically different from that of the CST group even though the exercise intensity for the MET group was only at 30% MVC level. The CTRL group did not have significant strength changes. Surprisingly, only the MET group demonstrated a significant augmentation in the MRCP (29.3%, *P* <0.001); the MRCP increase in CST group was at boarder-line significance level (12.11%, *P* = 0.061) and that for CTRL group was only 4.9% (*P* = 0.539). These results suggest that high mental effort training combined with low-intensity physical exercise is an effective method for voluntary muscle strengthening and this approach is especially beneficial for those who are physically weak and have difficulty undergoing conventional strength training.

## 1 Introduction

Motor imagery (MI) is an active cognitive process during which the representation of a specific action is internally reproduced within working memory without any overt motor output.^[1]^ MI or mental practice of motor skills has been used for over 50 years to help promote motor learning when physical practice is not possible.^[2,3]^ More recently, investigators have proposed that MI of muscle actions may be an effective rehabilitation tool in neuromuscular rehabilitation.^[4]^ It has been reported that physical training and mental practice of a motor skill resulted in a similar amount of improvement in performance and a similar pattern of adaptation in the primary motor cortex in human participants.^[5,6]^ MI on its own has not always proven to be effective in improving motor performance^[7]^; however, in combination with physical practice, it has been shown to be more effective than physical practice alone.^[8,9]^

While the effects of MI training (MIT) on improving motor skill learning are well established,^[7,10]^ only a limited number of studies have been carried out to investigate how MIT can change muscle strength. Ranganathan et al^[11]^ demonstrated MIT-induced strength gains in finger and upper-arm muscles that accompanied an increase in the cortical signal directly related to planning and execution of strength-production muscle contractions. Similar findings of voluntary strength improvements by MIT in both distal and proximal muscles have been reported by other investigating groups^[12–18]^; however, see Herbert et al.^[19]^ These observations support the hypothesis that the descending command from the brain to the target muscle for maximal voluntary contraction (MVC) can be augmented by MIT alone, which in turn increases muscle strength by recruiting additional motor units and/or elevating activation level of the participating motor units.^[18]^

The effect of MIT on muscle strength has been evaluated under the condition of joint and muscle immobilization. In one study participants who performed MIT during the immobilization maintained strength with a significant increase in the EMG signal despite muscle atrophy; however, those in the control group who had the immobilization but did not do MIT exhibited both muscle atrophy and strength loss without the EMG increase.^[20]^ The enhancement of neural (EMG) signal in the MIT group clearly compensated for strength loss due to the atrophy.^[20]^ Similarly, Clark et al^[3]^ found that the group performed MIT during immobilization attenuated strength loss by 50% compared with the group that only underwent immobilization. These results provide evidence of the neural origin of strength gain that occurs before muscle hypertrophy, hence driving the motor units to a higher intensity and/or leading to the recruitment of motor units that remain otherwise inactive.^[21]^ As discussed by Folland and Williams^[22]^ that although further research is clearly required, the available evidence in the literature suggests that substantial increase in the strength of major ambulatory muscle groups can be made without physical activity and morphological adaptations. It is clear, however, that despite the lack of a definitive understanding of the processes underpinning the effect of MIT, strength gains have been achieved with this practice technique.^[21]^ Because MI of high-intensity muscle contraction for strength improvement simply involves high mental or motor effort (ME) for intended muscle contraction without physical exercise, we hereafter refer this type of training to ME training (MET).

Although evidence of significant strength improvement by MET in healthy young individuals is accumulating,^[12–18]^ effects of MET on muscle strengthening and associated brain-to-muscle signal adaptation in clinical and aging populations have not been investigated. Thus, the novelties of the study are composed of investigating these variables in aging. Practically, MET is a more attractive approach for muscle strengthening in motor function-impaired populations especially those with significant weakness as conventional training regimes,^[6]^ such as high-intensity weightlifting may be unsafe or too difficult for many of them to perform. Therefore, the purpose of this study was to explore the effect of MET combined with low-intensity muscle contraction on voluntary strength of elbow flexor muscles in older adults. The reasons to add low-intensity muscle exercise into the MET were that it is easier to perform MET without completely “shutting down” the target muscle; elderly people are associated with reduced ability to effectively perform pure motor imagery^[23,24]^; and from a rehabilitation point of view, MET together with muscle exercise training, albeit at low intensity, would be more beneficial to the neuromuscular system than performing MET alone.^[8,17]^ It was hypothesized that MET combined with low-level muscle activities would induce significant strength gains.

## 2 Methods

### 2.1 Subjects

Twenty-seven healthy elderly (age: 75 ± 7.9 yr, 8 women) volunteers were assigned into one of 3 groups: motor effort training (MET) group performed maximal effort of elbow flexion of the right arm combined with low-intensity (30% MVC) physical exercise (n = 10, 3 women); conventional strength training (CST) group performed voluntary contractions of right elbow flexion at 80% MVC (n = 10, 3 women); and no-practice control (CTRL, 2 women) group was not trained, but participated in all testing sessions (n = 7). The study was a 3 (groups) × 2 (before and after training measures) experimental design. The local Institutional Review Board approved the study and all participants gave their informed consent prior to participation.

### 2.2 Motor effort training

The MET lasted for 12 weeks with 5 training sessions per week (M-F). In each training session, participants performed 50 ME trials combined with low-intensity muscle exercise. In each trial, they flexed the right elbow joint at 30% MVC while at the same time mentally urged the forearm to push upward (elbow flexion) maximally against the force transducer used for the pretraining strength measurement. This type of ME accompanies significant elevation of blood pressure and heart rate (10), and is considered kinesthetic or internal imagery.^[18,25–27]^ Each trial lasted 5 seconds followed by a 5-second rest. After 25 trials of training, a 2-minute rest was provided before doing the next 25 trials. Although participants were told to maintain the physical exercise at 30% MVC, they did not have to precisely match the target level so that their attention was primarily focused on the ME task.

### 2.3 Conventional strength training

We wondered whether a greater strength improvement could be realized by conventional strength (high-intensity physical exercise) training (CST). Participants in CST group performed 50 trials of right elbow flexion at 80% MVC level in each training session for 60 sessions (M-F, 12 weeks). In each trial, they performed isometric contraction of right elbow flexion at the target level. The target force was displayed on an oscilloscope and they were asked to reach the target and maintain the elbow flexion force on the target for 5 seconds. The number of training sessions and trials, and trial duration performed were the same between the MET and CST groups. The 80% MVC training intensity was adjusted every 2 weeks based on subjects’ newer MVC force.

### 2.4 Force (strength) measurement

**Figure 1:**
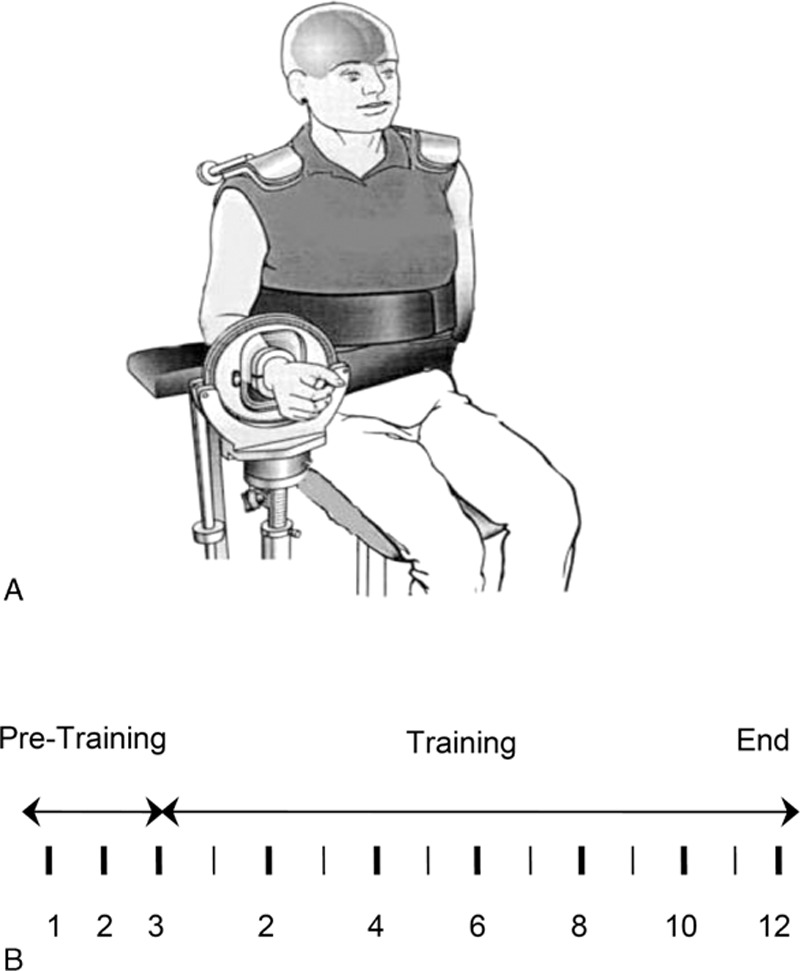
Experimental setup (A) and strength testing and training schedules (B). Vertical lines in (B) indicate time in weeks, with the thin lines representing odd numbers and thick lines even numbers in the training period. Each thick vertical line also indicates a strength measurement (during-training strength measures were used to adjust training intensity). Note there were 3 strength measuremnts in the 3 week-pretraining period to obtain a truce baseline strength value.

Elbow flexion force of the right arm was measured by a force transducer (JR3 Universal Force-Moment Sensor System, Woodland, CA) with subjects seated, their left hand placed in a wrist cuff, forearm in a neutral position, and an elbow joint angle of ∼100°.^[11]^ The elbow was supported at hip height and the shoulders and torso were kept in position using restraints (Fig. 1). Three MVC trials were performed in each measurement session and the highest force among the trials was analyzed. In each trial, participants were verbally encouraged to exert maximal force. Strength measurements were made before training and after the 12-week training period. The strength measurement conditions (arm and body positions, joint angles, etc.) were maintained across the evaluation sessions. In addition, the verbal instruction and encouragement for maximal force production were similar for all measurement sessions. The strength was measured 3 times in 3 separate sessions before training to ensure the pretraining strength was true maximal at the time.^[18]^ The elbow flexion force data collected during the strength measurements were digitized at 100 samples per second using a data acquisition system (Micro 1401; Cambridge Electronic Design, Ltd, Cambridge, UK) and recorded on hard disk of a personal computer (PC).

### 2.5 EMG measurement

Surface EMG was recorded during the elbow-flexion MVC force measurement trials using bipolar surface electrodes (Ag–Ag Cl; In Vivo Metric, Healdsburg, CA; 8-mm recording diameter and 2 cm apart of the 2 electrodes) from the belly of the biceps brachii (BB) and triceps brachii (TB) muscles. A reference electrode was placed on the skin overlying the lateral epicondyle near the elbow joint.^[18]^ The EMG signal was amplified (×1000) and band-pass filtered (3 Hz to 1 kHz) using a Neurodata Amplifier system (Model 15A54; Grass Instrument Co, Quincy, MA), digitized (2000 samples/s) using the Micro-1401 system, and recorded on hard drive of the PC. During offline processing/analysis, all the EMG signals were rectified (negative flipped to positive) before further analysis. For each muscle, the rectified EMG signal during a period when the MVC force was stable in each trial was averaged. The average BB MVC EMG was quantified as its absolute value in mV. The average TB EMG during the elbow flexion MVC was normalized to average TB EMG recorded during the elbow extension MVC and was a measure of the antagonist (TB) muscle activity during strength performance of the agonist (elbow flexor) muscle group. For the BB muscle, the MVC trial that yielded the highest average EMG was used for the analysis. For the TB, the value corresponded to the selected (highest) MVC EMG of the BB was used.

### 2.6 EEG and EEG-derived MRCP measurement

EEG electrodes were placed on the scalp roughly overlying the supplementary motor area (Cz), contralateral (C3), and ipsilateral (C4) sensorimotor regions, and central location of the frontal lobe (Fz). Electrode locations were determined based on the International 10-20 System. Conducting gel (Electro-gel; Electro-Cap International, Inc, Eaton, OH) was injected into each electrode to connect the recording surface of the electrode with the scalp.^[18]^ Impedance between each electrode and the skin was maintained below 5000 ohms. The EEG signal was amplified (×20,000, Model 15A54; Grass Instrument Co, Quincy, MA), band-pass filtered (0.1–100 Hz), digitized (300 samples/s) using the Micro-1401 system, and stored on hard disk of the PC.

The EEG data were acquired before and at the end of the 12-week training program. In each EEG session, participants performed 30 trials of the elbow flexion MVC. It is necessary to perform multiple MVC trials to improve the signal-to-noise ratio by trigger-averaging time-locked EEG epochs to obtain MVC-related cortical potential (MRCP). Raw EEG data were visually examined and trials with artifacts (such as eye blinks) were excluded. For each MVC trial, a 4-second window of the EEG was triggered by the force output (threshold = 5% initial MVC force), 2 second before, and 2 second after the trigger.^[11,28,29]^ The Spike 2 data analysis software (Cambridge Electronic Design, Ltd, Cambridge, UK) performed signal averaging over the 30 trials. The amplitude of each averaged MRCP was measured from the baseline to the peak of the negative potential.^[11,18,20,28,29]^ Because the MRCP was time-locked to each MVC, it was considered being directly related to the planning and execution of the MVC. Thus, increases in MRCP amplitude after training can be considered a direct indication of an enhancement in the descending command to the target muscle.

### 2.7 EEG power of frequency analysis

EEG power spectral analysis using fast Fourier transform (FFT) was performed on raw EEG data of each subject associated with the 3 trials of strength measurements before and after training. In each trial, a 2-second noise-free segment of EEG data was selected and a power spectrum (expressed as μV^2^) calculated. Consequently, the power for each of the following standard EEG frequency bands was derived: delta (0.5–4 Hz), theta (4–8 Hz), alpha (8–14 Hz), and beta (14–35 Hz).

### 2.8 Statistical analysis

To ensure that the baseline strength among the 3 groups was not different, the pretraining strength data were analyzed by one-way analysis of variance (ANOVA). There was no significant difference in the pretraining MVC force among the groups. Due to the repeated nature of the measurements of the MVC force, EMG and EEG data before and after training, a repeated measures ANOVA was employed for the within- and between-group comparisons. The level of significance was set at 0.05 for all statistical analyses. All data presented below were means ± standard errors (se).

## 3 Results

### 3.1 Strength gains were significant in both MET and CST groups

**Figure 2:**
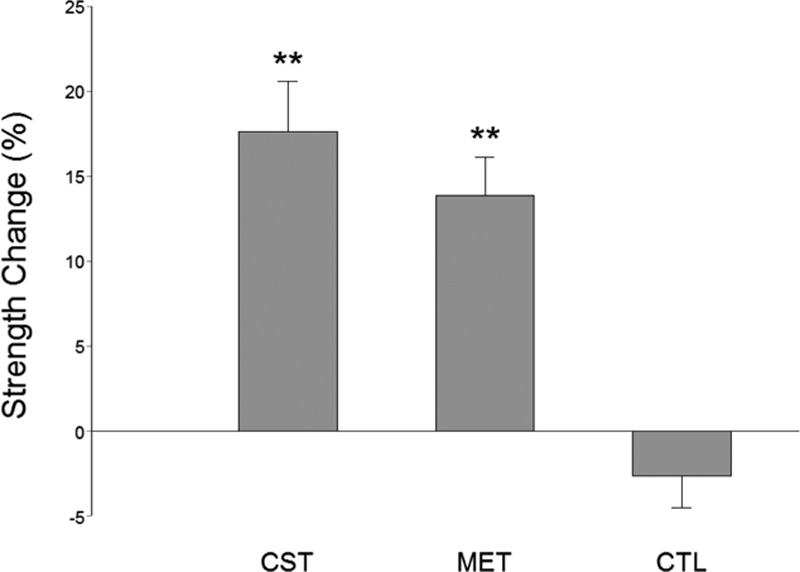
Percent elbow flexion strength changes in conventional (CST), motor effort (MET), and no-training control (CTL) groups following a 12-week training program. There was no significant difference in pretraining strength among the 3 groups. Both the CST and MET groups had significant strength gains after training. The CTL group did not have significant strength increase. ^∗∗^*P* <0.01.

After the 12-week training program, both MET and CST groups had strength gains compared with the baseline (Fig. 2). There was no significant difference in pretraining strength among the groups (*P* = 0.77). The CST group had the highest strength gain (17.58 ± 2.94%, *P* <0.001). The MET group also had significant strength gains (13.83 ± 2.26%, *P* <0.001), which was statistically similar to that of the CST group even though the exercise intensity for the MET was only 30% of MVC level. The CTL group did not have significant strength changes (Fig. 2). The absolute strength values (in Newton [N]) before and after training were 165.1 ± 12.9 and 193.7 ± 15.0 (CST, a 28.6 increase); 175.2 ± 16.1 and 201.3 ± 22.1 (MET, a 26.1 increase); and 182.9 ± 22.4 and 176.9 ± 21.5 (CTL, a 6.0 decrease), respectively.

### 3.2 EMG signals showed insignificant increases after training

The average EMG (AEMG) for the biceps brachii (BB) increased for both the CST and MET groups by 9.47 ± 10.82% and 32.26 ± 27.18%, respectively, but these changes did not reach statistical significance (*P* >0.05) primarily due to large intersubject variation in the data. BB AEMG remained similar before and after training for the CTL group (5.30 ± 10.55%). The antagonist muscle (triceps brachii) EMG during elbow flexion MVC was normalized to triceps MVC EMG during elbow extension MVC. No significant changes were found in the normalized antagonist muscle EMG in any of the groups after training.

### 3.3 MRCP increased as a result of MET

**Figure 3:**
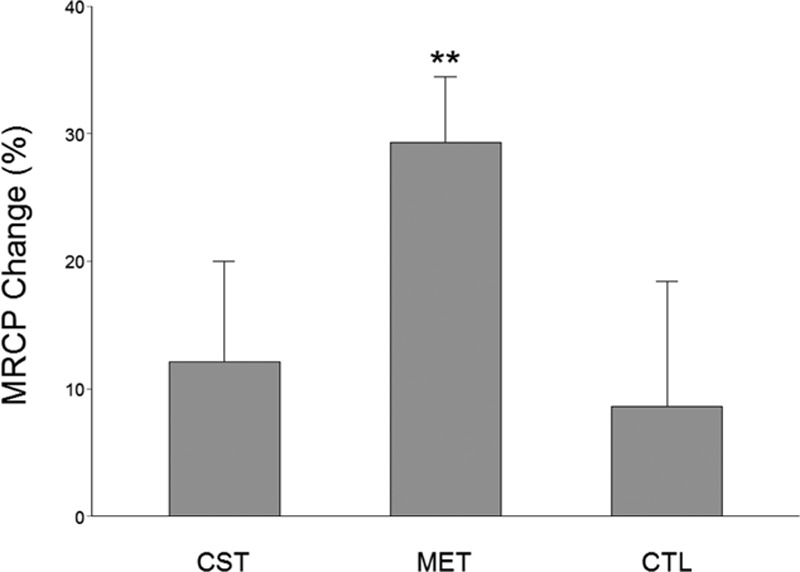
Percent MRCP (at Cz recording site) before and after the training program. Compared with the pretraining values, the MVC-related MRCP increased significantly for the MET group at the end of the 12 weeks of training. Even though the strength gain was highest for the CST group, the MRCP increase only attained boarder-line significance (*P* = 0.061). ^∗∗^*P* <0.01.

On average, the MVC-related MRCP values had no significant difference among the 3 groups before training (*P* = 0.15). Compared with the pretraining values, the MRCP (at Cz location) increased significantly for the MET group (29.30 ± 5.19%, *P* <0.001) at the end of the 12-week training (Fig. 3). (Because the Cz location exhibited greatest MRCP changes, here we focus on presenting MRCP data derived from this location.) Although the strength gain was slightly higher for the CST group, the MRCP increase in this group was less (12.11 ± 7.88%, *P* = 0.061) compared with the MET group (Fig. 3). The amount of increase in MRCP in MET group was significantly greater than that in CST group (*P* <0.01). The change in MRCP was not different between CST and CTL groups (*P* = 0.57). Because the MRCP was time locked to the MVC task, its increase implies strengthened cortical drive to the target muscle.

### 3.4 EEG frequency power increased as a result of MET

**Figure 4:**
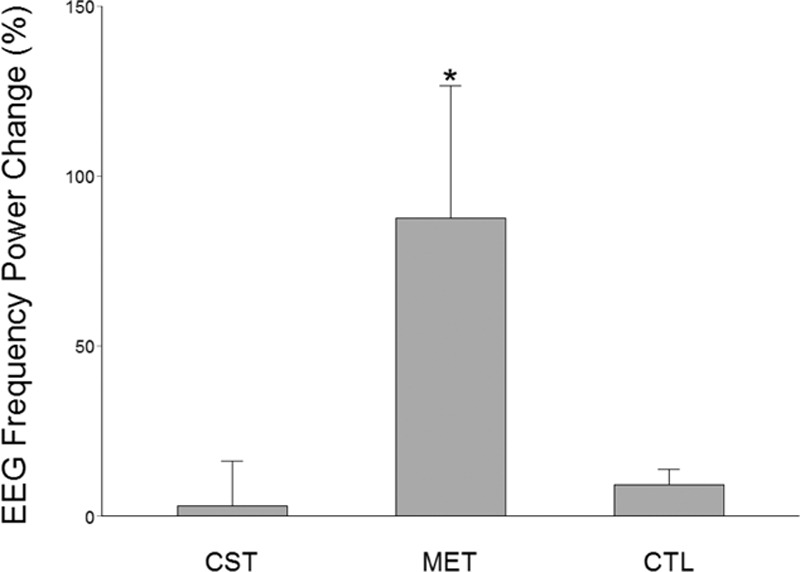
EEG frequency (alpha band, 8–13 Hz) power changes after versus before the 12-week training program at Cz location. Only the MET group exhibited a significant increase in the EEG power at this frequency band and recording location. ^∗^*P* <0.05.

**Figure 5:**
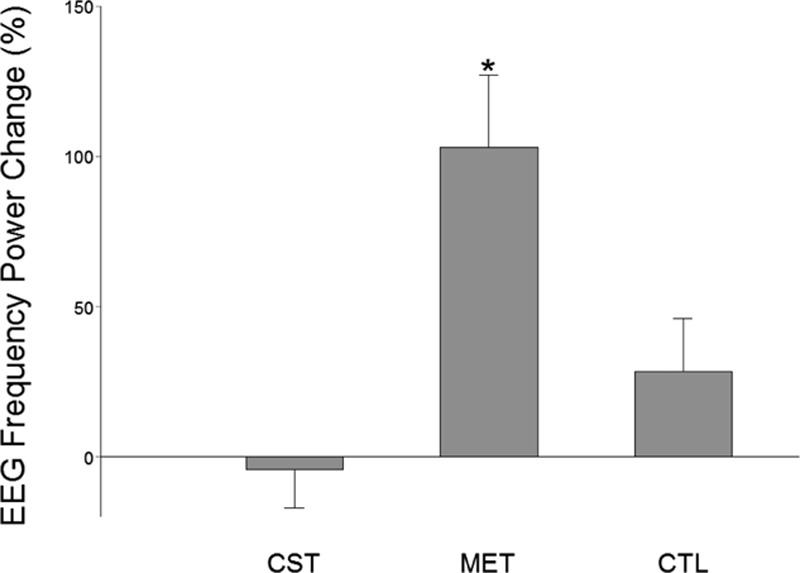
EEG frequency (alpha band, 13–35 Hz) power changes after versus before the 12-week training program at Cz location. Only the MET group exhibited a significant increase in the EEG power at this frequency band and recording location. ^∗^*P* <0.05.

**Figure 6:**
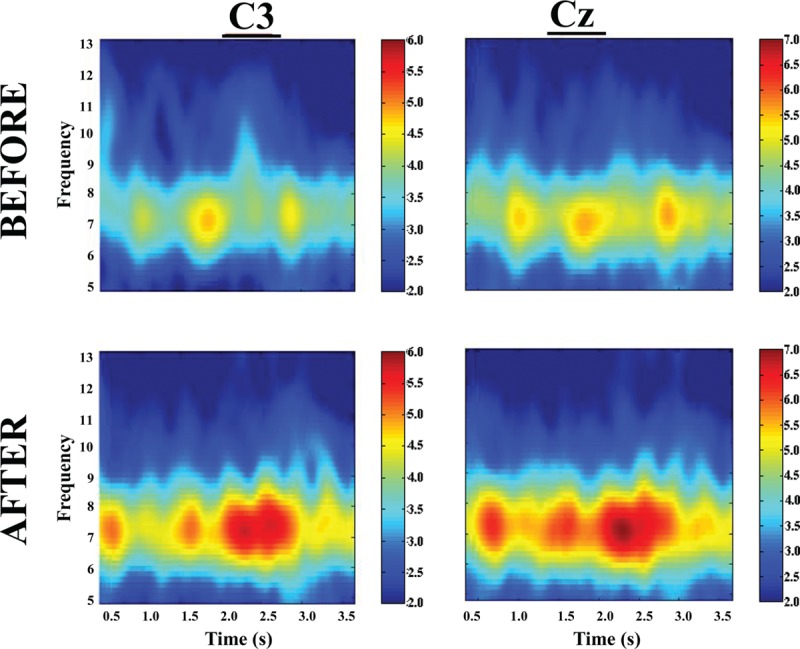
An example of time–frequency–power plots for a subject in the CST group before (top) and after (bottom) training at C3 (left) and Cz (right) recording locations. In each plot, the y-axis indicates EEG frequency and x-axis time points during the MVC trial with time 0 (not shown) depicting beginning of the trial, and color bar on right represents the power scales (red = greater power). Note a clear increase in power at high theta (7–8 Hz) frequency after training.

EEG frequency power has previously been shown to be related to motor activities.^[30]^ In particular, EEG frequency power has been found to be proportionally related to muscle contraction force.^[31]^ In this study, we found that power of EEG frequency increased significantly in both the CST and MET groups. However, only the MET group had significant power increases at both alpha (8–14 Hz, Fig. 4) and beta (14–35 Hz, Fig. 5) bands at the Cz (over supplementary motor area, Fig. 4) location. The CST group showed a significant power elevation (54.61 ± 29.39%, *P* <0.05) at theta band (4–8 Hz) at Cz location (not shown). The CTL group experienced no significant EEG frequency power alterations. Fig. 6 shows an example of enhanced EEG power of frequency at high theta or low alpha (7–8 Hz) as a function of time for the strength measurement MVC trials of a subject in CST group before and after training. For each plot in Fig. 6 the y-axis indicates EEG frequency and x-axis time points during the MVC trial with time 0 (not shown) depicting beginning of the trial, and color bar on right represents the power scales (red = greater power). It is clear that in this individual, the theta (4–8 Hz) frequency power increased substantially after the MET training at both C3 (left) and Cz (right) locations.

## 4 Discussion

The major findings of this study are strength of elbow flexor muscles, a frequently used large upper extremity muscle group during daily living, can be significantly improved by training of high motor effort combined with low-intensity muscle contraction in older adults, and the strength increase accompanied central signal augmentation that suggests an enhancement of descending command that was thought to have improved motor unit recruitment and activation leading to greater force production without changes in muscle morphology.^[17]^ The current study is the first to show MET-induced strength gains in aging population. The training regime (MET plus low-level muscle exercise) is an attractive approach for frail elderly individuals and weak patients to strengthen their muscles as it reduces difficulties and potential danger of injury involved in performing conventional high-intensity strength training, and difficulties associated with doing pure motor imagery as this involves dual processes of commanding (by the brain) strong motor activity but at the same time inhibiting it from happening physically. The finding that the difference in strength increases between the MET and CST groups was statistically insignificant argues that training of motor imagery combined with low-intensity muscle exercise is a safe and effective method for muscle strengthening for vulnerable populations such as frail older individuals.

### 4.1 Mechanism contributing to the MET-induced strength gains in the elderly

As expected, conventional training group (CST) which trained at 80% MVC intensity had the greatest strength gain, albeit the extra gain relative to that of the MET group was not statistically significant. (Strength training at about 80% maximal intensity that can be repeated ∼10 times with best effort [e.g., lifting a weight 10 times that equals 80% maximal weight that can be lifted] without rest and perform few sets of the 10-trial set in each training session yields the best result in strength gain.^[32]^) Our results indicate that, in the elderly population, when low-intensity exercises were combined with strong mental effort, the strength gain seen was similar to that of high-intensity exercises. Based on the MRCP data (Fig. 3) and power of EEG frequency results (Figs. 4–6), we are confident that the primary mechanism contributed to the strength increase is MET-induced enhancement in the central command to muscle. The data suggest that repetitive mental attempts of maximal muscle activation trained and enabled motor network in the brain to generate stronger signal to muscle. Previous research has shown a proportional relationship between magnitude of brain-to-muscle signal (MRCP) and voluntary muscle force by young human subjects, indicating that greater strength is a consequence of stronger brain-to-muscle signal.^[28]^ A recent study in aging demonstrated that corticomuscular signal coupling measured by EEG-EMG coherence at beta frequency band (15–35 Hz) correlates significantly with elbow flexion force, further suggesting a direct relationship between cortical motor command and force output of muscle.^[33]^ The MRCP and EEG frequency power results in this study suggest MET can induce similar functional adaptations in the brain in elderly people compared with those seen in young.^[11,18]^ Changes in muscle coordination (i.e., a reduction in antagonist muscle activation during the strength measurement as suggested by ^[34]^) did not occur and was not a significant factor contributing to the strength increase in the MET group.

It is not clear what forms of neural adaptations occurred and at what levels in the CNS resulted from the motor effort training. It is unlikely that the adaptations occurred in subcortical centers because the training primarily targeted the cerebral cortex (strong voluntary effort elbow flexions). We argue that the mental effort exercise primarily trained higher-order cortical regions that could influence cortical motor output to the target muscle. Repetitive attempts of maximal activation during training may change synaptic strength between high-order cortical areas and the primary motor cortex (M1) projecting directly to motoneuron pool of the target muscle, thereby improving excitability of the M1 so that when MVCs were performed during the post-training test session, a greater number of output neurons in the M1 may be recruited and/or their activation pattern may be changed (e.g., increases in firing rate and level of synchronization) to increase the output signal. The most probable higher-order cortical centers that were trained by the high-effort contractions are secondary and association motor cortices such as supplementary motor^[35]^ and prefrontal^[36]^ areas.

### 4.2 Explanation for the similarity in strength gains between the CST and MET groups

The CST group had the highest strength gain (17.6%, *P* <0.001). On the other hand, the MET group also improved strength significantly (13.8%, *P* <0.001), which was statistically similar to that of the CST group even though the exercise intensity was only at 30% MVC level. A greater strength increase was expected for the CST group given the expectation that subjects in this group were to experience both neural and muscular adaptations that were likely contribute to greater muscle (force) output while primarily only neural changes were expected in the MET group. An explanation for this could be that the MET subjects achieved stronger neural signal (descending command) increase than those who were trained by CST as the MET participants were more focused on training the central nervous system and this may have benefited the neural adaptation more than the high-intensity physical training. If this was true, then a greater strength gain resulted from a stronger descending command would have compensated for a smaller increase in MVC force due to lack of a significant muscle mass increase in the MET group. The larger MRCP gain in the MET group in the current study and a previous report^[11]^ seems to support this assumption. The observation of greater changes in EEG frequency power in the MET than CST groups (Figs. 4–6) also supports the argument.

### 4.3 Explanation for insignificant EMG results

The EMG value changes in both the MET and CST groups did not reach significance level following the training program. Because the EMG signals reflect motor unit activities during a muscle contraction, a significant increase in the EMG signal was expected if motor unit activity (recruitment and firing rate) level was elevated after training. However, surface EMG values are influenced by many factors besides motor unit action potentials, including locations of the recording electrode, positions of the electrodes relative to muscle fiber orientation, mass and quality of tissues between the electrode and muscle, and skin impedance.^[37]^ One or more of these factors may have changed from the pre- to post-training measurement sessions despite our effort to keep them as close being the same as possible and they may have masked true EMG signal enlargement due to improved motor unit activities. Previous MET studies found significant increases in surface EMG that was normalized to the maximal evoked compound action potential (M wave) collected from the same muscle and session (Yue and Cole^[13]^ [EMG of abductor digiti minimi normalized to M wave by electrically stimulating ulnar nerve] and Ranganathan et al^[11,38,39]^ [EMG of brachiradialis normalized to M wave by simulating radial nerve]). It is difficult to locate the nerve supplying the biceps brachio muscle from the surface for stimulating and recording the M wave.

## 5 Limitations

A major limitation of the study was that muscle mass of the elbow flexors was not measured. This limited our ability to estimate the amount of strength gain from increases in muscle size and that from the neural adaptation, especially for the CST group.

## 6 Conclusion

Low-intensity (30% maximal voluntary contraction [MVC]) physical exercise combined with strong motor effort training could help elderly people effectively strengthen their frequently used and functionally important muscles for everyday life. The motor effort training-induced central nervous system signal augmentation is considered being primarily responsible for the strength gain. The findings of increases in motor-related cortical potential and EEG frequency power associated with maximal voluntary contractions following the motor effort training are significant steps forward for a better understanding of neural mechanisms underlying voluntary muscle strengthening in general and in aging population in particular. Our findings could potentially lead to development of effective and economical rehabilitation interventions for treating muscle weakness.
